# Blindness and Legislation

**Published:** 1906-12

**Authors:** F. Park Lewis

**Affiliations:** 454 Franklin Street. Buffalo


### "Blindness and Legislation."

**Dr. F. Park Lewis answers Criticisms in Regard to proposed Legislation looking to the Prevention of Ophthalmia Neonatorum.**

EDITOR BUFFALO MEDICAL JOURNAL.

SIR: The following editorial paragraph is clipped from the October issue of the Saint Louis Courier of Medicine, which I desire a little space in your journal to answer, as it seems important that a clear understanding of this whole matter shall be had.

"BLINDNESS AND LEGISLATION.

"Does it not seem strange that legislation should be con-"sidered necessary to urge physicians and midwives to do "their duty? At least that is what the resolution of Park "Lewis at the Boston meeting of the American Medical As-"sociation really means. We question very much that blind-"ness due to ophthalmia will become very much reduced by "such legislation. The disease is certainly much less dread-"ed now than formerly, and Crédè's method is more gener-"ally used; consequently, an additional legislative act seems "an unnecessary burden on the statute books. It is well, "however, for the Boards of Health to add ophthalmia neon-"atorum to the list of contagious diseases; it will make the "physicians more circumspect if they are compelled to report "these cases.—*St. Louis Courier of Medicine,* October."

The above would seem to be a very natural criticism on a most important subject and as the misapprehensions on which it is based may be shared by others, may I be permitted space to correct them? As Crédè and many others have demonstrated, the use of silver nitrate is an almost absolute preventive and specific for ophthalmia neonatorum. If some form of silver is not employed this disease is quite as prevalent and quite as dangerous as it has ever been. While it is true that in the better hospitals and in the hands of many careful physicians the Crédè method has become a routine measure,—there are still large numbers of practising physicians who never use it until the ophthalmia has developed, if they do then,—when it is often too late; while by the thousands of midwives it is never used at all. Some years ago I personally investigated the number of births attended by midwives in the City of Buffalo for a single year and I found the proportion to be more than one half. About nine thousand children were born in this city in 1900. No midwife has, or is expected, to use the Crédè silver solution as a part of the child's toilet. It does not occasion surprise, therefore, that cases of infantile ophthalmia are still appearing in the clinics at ophthalmic hospitals and that a procession of sightless children is still annually augmenting the ranks of our schools for the blind.

The pathos of this needless blindness must appeal to every humane instinct of those who witness it. The blot on our medical scutcheon, due to our apathy in allowing a preventable germ disease for which the specific is known to continue and to result in a prodigal waste of human eyes year after year, is a challenge to our claim as a scientific body. This disease as a cause of blindness should and must be absolutely wiped out.

The method by which this measure of relief can be applied is simple. Legislation cannot compel the use of a medical measure but it can make possible its employment. Doubtless these hundreds of good women practising their vocations as accoucheurs would gladly use the silver solution in the new-born babies' eyes but they have not got it. Even if permitted, as they are not, they could not write a prescription for it and they would not know whether the nitrate of silver should be used in a 2% or in a 50% solution.

There is but one way in which the routine employment of the silver salt as a part of the infant's toilet may be assured and that is to put in the hands of the accoucheur, whether midwife or physician, *a sealed receptacle, containing a pure solution, of ascertained strength, with directions for its employment,* the whole with the authority and by the advice of the State or local Board of Health.

This makes imperative uniform legislation which can be secured only by the hearty cooperation of the medical press. The legislation required in any state is that which will place by statute ophthalmia neontorum on the list of infectious communicable diseases—which will secure an annual appropriation of sufficient size to permit the local health officers to distribute gratuitously a preparation of the silver wherever necessary and to take such other measures as may be required to give sanitary protection to the eyes of every new-born child.

454 Franklin Street. F. Park Lewis.
Buffalo, November 5, 1906.

